# Supplementary material for: A Systematic Review on the Role of Substance Consumption in Work-Related Road Traffic Crashes Reveals the Importance of Biopsychosocial Factors in Prevention
Source: Behav Sci (Basel). 2022 Jan 25;12(2):23. doi: 10.3390/bs12020023 (PMC8869722; doi:10.3390/bs12020023)
Supplement: Supplementary file 1 [file behavsci-12-00023-s001.zip › Supplementary Materials.pdf]

SUPPLEMENTARY MATERIALS

Supplementary Table S1: PRISMA Check-List (Moher et al., 2009; Page et al.,2021) [NEAR HERE]

Supplementary Table S2: Appraisal tool for Cross-Sectional Studies (AXIS; Downes et al., 2016) [NEAR HERE]

Supplementary Table S3: PRISMA Abstract Check-List (Page et al.,2021) [NEAR HERE]

Supplementary Table S4: Study Research [NEAR HERE]

Supplementary references

Downes, M. J., Brennan, M. L., Williams, H. C., & Dean, R. S. (2016). Development of a critical appraisal tool to assess the quality of cross-sectional studies (AXIS). *BMJ Open*, *6*(12), e011458. https://doi.org/10.1136/bmjopen-2016-011458

Moher, D., Liberati, A., Tetzlaff, J., Altman, D. G., & PRISMA Group. (2009). Preferred reporting items for systematic reviews and meta-analyses: The PRISMA statement. *PLoS Medicine*, *6*(7), e1000097. https://doi.org/10.1371/journal.pmed.1000097

Page, M. J., McKenzie, J. E., Bossuyt, P. M., Boutron, I., Hoffmann, T. C., Mulrow, C. D., ... & Moher, D. (2021). The PRISMA 2020 statement: an updated guideline for reporting systematic reviews. *Bmj*, *372 :n71*. doi:10.1136/bmj.n71
