# Supplementary material for: A Systematic Review on the Role of Substance Consumption in Work-Related Road Traffic Crashes Reveals the Importance of Biopsychosocial Factors in Prevention
Source: Behav Sci (Basel). 2022 Jan 25;12(2):23. doi: 10.3390/bs12020023 (PMC8869722; doi:10.3390/bs12020023)
Supplement: Supplementary file 1 [file behavsci-12-00023-s001.zip › Supplementary Table S2_AXIS tool.pdf]

The Appraisal tool for Cross-Sectional Studies (AXIS tool, 2016) Part 1							
Study	**Asefa et al., 2015**	**Bacchieri et al., 2010**	**Bamberger and Cohen, 2015**	**Boufous and Williamson, 2006**	**Chu, 2014**	**Karakus et al., 2015**	**Sam et al., 2018**
Introduction							
1. Were the aims/objectives of the study clear?	YES	YES	YES	YES	YES	YES	YES
Methods							
2. Was the study design appropriate for the stated aim(s)?	YES	YES	YES	YES	YES	YES	YES
3. Was the sample size justified?	YES	YES	NO	NO	NO	NO	NO
4. Was the target/reference population clearly defined? (Is it clear who the research was about?)	YES	YES	YES	YES	YES	YES	YES
5. Was the sample frame taken from an appropriate population base so that it closely represented the target/reference population under investigation?	YES	YES	YES	YES	YES	YES	YES
6. Was the selection process likely to select subjects/participants that were representative of the target/reference	YES	YES	NO	YES	YES	YES	YES

population under investigation?							
7. Were measures undertaken to address and categorise non-responders?	NO	NO	YES	NO	NO	NO	NO
8. Were the risk factor and outcome variables measured appropriate to the aims of the study?	YES	YES	YES	YES	YES	NO	YES
9. Were the risk factor and outcome variables measured correctly using instruments/ measurements that had been trialled, piloted or published previously?	YES	YES	YES	YES	YES	YES	YES
10. Is it clear what was used to determined statistical significance and/or precision estimates? (eg, p values, CIs)	YES	YES	YES	YES	YES	YES	YES
11. Were the methods (including statistical methods) sufficiently described to enable them to be repeated?	YES	YES	YES	NO	YES	NO	YES
Results							
12. Were the basic data adequately described?	YES	YES	YES	YES	YES	YES	YES

13. Does the response rate raise concerns about non-response bias?	NO	NO	YES	NO	NO	NO	YES
14. If appropriate, was information about non-responders described?	NO	NO	YES	NOT APPROPRIATE	NOT APPROPRIATE	NOT APPROPRIATE	NO
15. Were the results internally consistent?	YES	YES	YES	YES	YES	YES	YES
16. Were the results for the analyses described in the methods, presented?	YES	YES	YES	YES	YES	YES	YES

**The Appraisal tool for Cross-Sectional Studies (AXIS tool, 2016) Part 2**							
Study	**Smailović et al., 2019**	**Thiese et al., 2015**	**Thygerson et al., 2011**	**Chen et al., 2020**	**Konlan et al., 2020**	**French and Gumus, 2021**	**Mitchell et al.,2014**
Introduction							
1. Were the aims/objectives of the study clear?	YES	YES	YES	YES	YES	YES	YES
Methods							
2. Was the study design appropriate for the stated aim(s)?	YES	YES	YES	YES	YES	YES	YES
3. Was the sample size justified?	NO	NO	NO	NO	YES	NO	NO
4. Was the target/reference	YES	YES	YES	YES	YES	YES	YES

population clearly defined? (Is it clear who the research was about?)							
5. Was the sample frame taken from an appropriate population base so that it closely represented the target/reference population under investigation?	YES	NO	YES	YES	YES	YES	YES
6. Was the selection process likely to select subjects/participants that were representative of the target/reference population under investigation?	YES	YES	YES	YES	YES	YES	YES
7. Were measures undertaken to address and categorise non-responders?	NO	NO	NO	NO	NO	NO	NO
8. Were the risk factor and outcome variables measured appropriate to the aims of the study?	YES	YES	YES	YES	YES	YES	YES
9. Were the risk factor and outcome variables measured correctly using instruments/ measurements that had been trialled, piloted or published previously?	YES	NO	YES	YES	YES	YES	YES

10. Is it clear what was used to determined statistical significance and/or precision estimates? (eg, p values, CIs)	YES	YES	YES	YES	YES	YES	YES
11. Were the methods (including statistical methods) sufficiently described to enable them to be repeated?	YES	YES	YES	YES	NO	YES	NO
Results							
12. Were the basic data adequately described?	YES	YES	YES	YES	YES	YES	YES
13. Does the response rate raise concerns about non-response bias?	NO	NO	NO	NO	NO	NO	NO
14. If appropriate, was information about non-responders described?	NOT APPROPRIATE	NO	NOT APPROPRIATE	NOT APPROPRIATE	NO	NOT APPROPRIATE	NOT APPROPRIATE
15. Were the results internally consistent?	YES	YES	YES	YES	YES	YES	YES
16. Were the results for the analyses described in the methods, presented?	YES	YES	YES	YES	YES	YES	YES

The Appraisal tool for Cross-Sectional Studies (AXIS tool, 2016) Part 3							
Study	**Poku et al.,2020**	**Brodie et al., 2009**	**Holizki et al., 2015**	**Lambrechts et al., 2019**	**Rudisill et al., 2019**	**Yuan, 2021**	**Li et al.,2020**
Introduction							
1. Were the aims/objectives of the study clear?	YES	YES	YES	YES	YES	YES	YES
Methods							
2. Was the study design appropriate for the stated aim(s)?	YES	YES	YES	YES	YES	YES	YES
3. Was the sample size justified?	YES	NO	NO	NO	NO	NO	NO
4. Was the target/reference population clearly defined? (Is it clear who the research was about?)	YES	YES	YES	YES	YES	YES	YES
5. Was the sample frame taken from an appropriate population base so that it closely represented the target/reference population under investigation?	YES	YES	YES	NO	YES	YES	YES
6. Was the selection process likely to select subjects/participants that were representative of the target/reference population under investigation?	YES	YES	YES	YES	YES	YES	YES
7. Were measures undertaken to address and categorise non-responders?	NO	NO	YES	NO	YES	NO	NO

8. Were the risk factor and outcome variables measured appropriate to the aims of the study?	YES	YES	YES	YES	YES	YES	YES
9. Were the risk factor and outcome variables measured correctly using instruments/ measurements that had been trialled, piloted or published previously?	YES	YES	YES	YES	YES	YES	YES
10. Is it clear what was used to determined statistical significance and/or precision estimates? (eg, p values, CIs)	YES	NO	YES	YES	YES	YES	YES
11. Were the methods (including statistical methods) sufficiently described to enable them to be repeated?	NO	NO	YES	YES	YES	YES	YES
Results							
12. Were the basic data adequately described?	YES	YES	YES	YES	YES	YES	YES
13. Does the response rate raise concerns about non-response bias?	NO	NO	YES	YES	YES	NO	NO
14. If appropriate, was information about non-responders described?	NO	NOT APPROPRIATE	YES	NO	NO	NOT APPROPRIATE	NOT APPROPRIATE
15. Were the results internally consistent?	YES	YES	YES	YES	YES	YES	YES

16. Were the results for the analyses described in the methods, presented?	YES	YES	YES	YES	YES	YES	YES

**The Appraisal tool for Cross-Sectional Studies (AXIS tool, 2016) Part 4**									
Study	**Liu & Fan, 2020**	**Papalia et al., 2012**	**Bourdeau et al., 2021**	**McNeilly et al., 2010**	**Qi et al., 2013**	**Gates et al., 2013**	**Wadsworth et al., 2006**	**Khoshakhlagh et al., 2019**	**Reguly et al., 2014**
Introduction									
1. Were the aims/objectives of the study clear?	YES	YES	YES	YES	YES	YES	YES	YES	YES
Methods									
2. Was the study design appropriate for the stated aim(s)?	YES	YES	YES	YES	YES	YES	YES	YES	YES
3. Was the sample size justified?	NO	NO	NO	NO	NO	NO	NO	NO	NO
4. Was the target/reference population clearly defined? (Is it clear who the research was about?)	YES	YES	YES	YES	YES	YES	NO	YES	YES

5. Was the sample frame taken from an appropriate population base so that it closely represented the target/reference population under investigation?	YES	NO	YES	YES	YES	YES	NO	NO	YES
6. Was the selection process likely to select subjects/participants that were representative of the target/reference population under investigation?	YES	NO	YES	YES	YES	YES	YES	NO	YES
7. Were measures undertaken to address and categorise non-responders?	NO	NO	NO	NO	YES	NO	NO	NO	NO
8. Were the risk factor and outcome variables measured appropriate to the aims of the study?	YES	YES	YES	YES	YES	YES	YES	YES	YES
9. Were the risk factor and outcome variables measured	YES	YES	YES	YES	YES	YES	YES	NO	YES

correctly using instruments/ measurements that had been trialled, piloted or published previously?									
10. Is it clear what was used to determined statistical significance and/or precision estimates? (eg, p values, CIs)	YES	YES	YES	NO	YES	YES	YES	YES	YES
11. Were the methods (including statistical methods) sufficiently described to enable them to be repeated?	YES	NO	YES	NO	YES	YES	YES	NO	YES
Results									
12. Were the basic data adequately described?	YES	YES	YES	YES	YES	YES	YES	YES	YES
13. Does the response rate raise concerns about non-response bias?	NO	NO	NO	NO	YES	NO	NO	NO	NO
14. If appropriate, was information about non-	NOT APPROPRIAT E	NO	NOT APPROPRIAT E	NOT APPROPRIAT E	YES	NOT APPROPRIAT E	NO	NO	NOT APPROPRIAT E

responders described?									
15. Were the results internally consistent?	YES	YES	YES	YES	YES	YES	YES	YES	YES
16. Were the results for the analyses described in the methods, presented?	YES	YES	YES	YES	YES	YES	YES	YES	YES
