# Supplementary material for: A Systematic Review on the Role of Substance Consumption in Work-Related Road Traffic Crashes Reveals the Importance of Biopsychosocial Factors in Prevention
Source: Behav Sci (Basel). 2022 Jan 25;12(2):23. doi: 10.3390/bs12020023 (PMC8869722; doi:10.3390/bs12020023)
Supplement: Supplementary file 1 [file behavsci-12-00023-s001.zip › Supplementary Table S4_Study Research.pdf]

Database	Steps	Query	Research in	Items found
**PubMed**	#1	alcohol OR amphetamine OR analgesic OR antidepressant OR antihistamine OR anxiolytic OR BAC OR barbiturate OR benzodiazepine OR cannabinoid OR cannabis OR cocaine OR drink OR drink-drive OR drink-driving OR " drinking AND driving" OR "driving under the influence" OR "driving while intoxicated" OR " driving while intoxicated" OR drug OR drunk OR drunk driving OR drunk-driving OR DUI OR DUID OR etanhol OR heroin OR hypnotic OR intoxicated OR medication OR narcotic OR opiate OR opioid OR polypharmacy OR psychoactive OR psychostimulant OR psychotropic OR sedative OR stimulant OR substance OR tranquilizer	Title/Abstract	2180247
	#2	bus OR "business driver" OR "commercial driver" OR commuting OR "company car" OR "delivery worker" OR "emergency vehicle" OR "grey fleet" OR "heavy commercial" OR vehicle OR itinere OR job OR "light commercial vehicle" OR lorry OR "occupational driver" OR "private car" OR "professional driver" OR "public transport vehicle" OR taxi OR trailer OR transport OR transportation OR truck OR work OR workplace OR work-place OR work-related	Title/Abstract	1553034
	#3	accident OR blameworthiness OR collision OR crash OR crashes OR culpability OR death OR fatalities OR fatality OR incident OR injuries OR injury OR "near miss" OR "road risk"	Title/Abstract	1709287
	#4	Combine #1 AND #2 AND #3		8897
	#5	Limit to (English) and (Italian)		8460
	#6	Limit to (year 2006- year 2021)		6594
**Scopus**	#1	alcohol OR amphetamine OR analgesic OR antidepressant OR antihistamine OR anxiolytic OR BAC OR barbiturate OR benzodiazepine OR cannabinoid OR cannabis OR cocaine OR drink OR drink-drive OR drink-driving OR " drinking AND driving" OR "driving under the influence" OR "driving while intoxicated" OR " driving while intoxicated" OR drug OR drunk OR drunk driving OR drunk-driving OR DUI OR DUID OR etanhol OR heroin OR hypnotic OR intoxicated OR medication OR narcotic OR opiate OR opioid OR polypharmacy OR psychoactive OR psychostimulant OR psychotropic OR sedative OR stimulant OR substance OR tranquilizer	Title/Abstract/ Keywords	10408145
	#2	bus OR "business driver" OR "commercial driver" OR commuting OR "company car" OR "delivery worker" OR "emergency vehicle" OR "grey fleet" OR "heavy	Title/Abstract/ Keywords	210147

		commercial" OR vehicle OR itinere OR job OR "light commercial vehicle" OR lorry OR "occupational driver" OR "private car" OR "professional driver" OR "public transport vehicle" OR taxi OR trailer OR transport OR transportation OR truck OR work OR workplace OR work-place OR work-related		
	#3	accident OR blameworthiness OR collision OR crash OR crashes OR culpability OR death OR fatalities OR fatality OR incident OR injuries OR injury OR "near miss" OR "road risk"	Title/Abstract/ Keywords	4096890
	#4	Combine #1 AND #2 AND #3		881
	#5	Limit to (English) and (Italian)		790
	#6	Limit to (from year 2006 to year 2021)		519

TABLE 2|Study research
